# Direct conversion of mouse astrocytes into neural progenitor cells and specific lineages of neurons

**DOI:** 10.1186/s40035-018-0132-x

**Published:** 2018-11-05

**Authors:** Kangmu Ma, Xiaobei Deng, Xiaohuan Xia, Zhaohuan Fan, Xinrui Qi, Yongxiang Wang, Yuju Li, Yizhao Ma, Qiang Chen, Hui Peng, Jianqing Ding, Chunhong Li, Yunlong Huang, Changhai Tian, Jialin C. Zheng

**Affiliations:** 1 Center for Translational Neurodegeneration and Regenerative Therapy, Shanghai Tenth People’s Hospital affiliated to Tongji University School of Medicine, Shanghai, 200072 China; 2 Collaborative Innovation Center for Brain Science, Tongji University, Shanghai, 200092 China; 3 Departments of Pharmacology and Experimental Neuroscience, University of Nebraska Medical Center, Omaha, NE 68198-5930 USA; 4 Department of Neurology & Institute of Neurology, Ruijin Hospital affiliated to Shanghai Jiao Tong University School of Medicine, Shanghai, 200025 China; 5 Department of Pathology and Microbiology, University of Nebraska Medical Center, Omaha, NE 68198-5930 USA

**Keywords:** Astrocytes, iNPCs, Reprogramming, Transcription factor, Neuronal lineage, Cholinergic neurons, Dopaminergic neurons, Lhx8, Foxa2, Lmx1a

## Abstract

**Background:**

Cell replacement therapy has been envisioned as a promising treatment for neurodegenerative diseases. Due to the ethical concerns of ESCs-derived neural progenitor cells (NPCs) and tumorigenic potential of iPSCs, reprogramming of somatic cells directly into multipotent NPCs has emerged as a preferred approach for cell transplantation.

**Methods:**

Mouse astrocytes were reprogrammed into NPCs by the overexpression of transcription factors (TFs) Foxg1, Sox2, and Brn2. The generation of subtypes of neurons was directed by the force expression of cell-type specific TFs Lhx8 or Foxa2/Lmx1a.

**Results:**

Astrocyte-derived induced NPCs (AiNPCs) share high similarities, including the expression of NPC-specific genes, DNA methylation patterns, the ability to proliferate and differentiate, with the wild type NPCs. The AiNPCs are committed to the forebrain identity and predominantly differentiated into glutamatergic and GABAergic neuronal subtypes. Interestingly, additional overexpression of TFs Lhx8 and Foxa2/Lmx1a in AiNPCs promoted cholinergic and dopaminergic neuronal differentiation, respectively.

**Conclusions:**

Our studies suggest that astrocytes can be converted into AiNPCs and lineage-committed AiNPCs can acquire differentiation potential of other lineages through forced expression of specific TFs. Understanding the impact of the TF sets on the reprogramming and differentiation into specific lineages of neurons will provide valuable strategies for astrocyte-based cell therapy in neurodegenerative diseases.

**Electronic supplementary material:**

The online version of this article (10.1186/s40035-018-0132-x) contains supplementary material, which is available to authorized users.

## Background

Neural progenitor cells (NPCs) exist throughout life and are able to proliferate and generate neurons, astrocytes, and oligodendrocytes in the central nervous systems (CNS) [1]. Recently, NPCs have also been shown to modulate immune response and protect neurons in the CNS [2, 3]. Due to their regenerative potentials, NPCs have been implicated in the treatment of neurodegenerative diseases [4, 5]. However, limited number and restricted anatomical locations have reduced the feasibility of using NPCs as a therapeutic approach. NPCs derived from human fetal tissues/embryonic stem cells may serve as an alternative cell source. However, this approach receives criticism over ethical concerns. The emerging field of cell reprogramming gives NPC-based therapy a boost because it avoids the aforementioned problems. Therefore, reprogrammed NPCs appear to be an attractive novel strategy for the treatment of neurodegenerative disorders [6–9].

Two common approaches are used to reprogram somatic cells into induced neural progenitor cells (iNPCs). The first one is to generate iNPCs through induced pluripotent stem cells (iPSCs), more primitive stem cells than NPCs [4, 10–17]. Because iPSCs derive from autologous tissues, the usage of iPSCs will prevent the deleterious immune reactions initiated by cell transplantation or replacement. However, iPSCs have also been noted to demonstrate variable potencies for neuronal differentiation as well as high risk of teratoma formation [4, 10], both of which pose challenges for clinical use. The second approach is the direct conversion of somatic cells into self-renewable and lineage-restricted NPCs by ectopic expression of defined transcription factors (TFs). Since 2011, we, along with other groups, have reported that defined TFs sets can reprogram somatic cells into iNPCs [13, 17–23]. Because the initial cell sources and the TFs sets used in these studies were not uniform, the resulting iNPCs showed variable NPC properties such as their distinct differentiation potentials for subtypes of neurons.

Because astrocytes are the most abundant type of cells within the CNS and reactive astrogliosis are present in all neurodegenerative disorders [24–26], astrocytes have become a key cell source for cell reprogramming [27]. Our previous data has suggested that astrocyte-derived iPSCs possess more tendencies for neuronal differentiation than fibroblast-derived iPSCs [17]. In the current studies, we used a set of TFs previously used for fibroblast-derived iNPC reprogramming [19]. We demonstrated that this set of TFs could successfully reprogram mouse astrocytes into tripotent iNPCs (AiNPCs). The AiNPCs expressed NPC-specific genes, acquired characteristic NPC functions, including self-renewal and proliferation. Interestingly, AiNPCs seemed to have a regional fate commitment with restricted forebrain differentiation competency. The subtype specification studies further suggested that the differentiation of AiNPCs is biased to favor the glutamatergic and GABAergic lineages over the dopaminergic lineage. Interestingly, the regional and lineage commitment of AiNPCs could be overwritten by additional overexpression of key TFs specific for neuronal subtype differentiation. For example, Lhx8 could induce cholinergic neuron differentiation and Foxa2/Lmx1a could induce midbrain dopaminergic neuronal differentiation. Together, our data provide further understanding on astrocyte-based cell reprogramming for the development of future therapies in neurodegenerative diseases.

## Methods

### Mice and astrocyte culture

E/Nestin:EGFP mice (kindly provided by Dr. Richard J. Miller from Northwestern University, Chicago, IL) are housed and bred in the Comparative Medicine animal facilities at the University of Nebraska Medical Center (UNMC). All procedures were conducted according to protocols approved by the Institutional Animal Care and Use Committee of the UNMC. E/Nestin:EGFP positive transgenic pups were identified by direct fluoresce visualization under a IVIS optical imaging system and validated by genotyping. Astrocytes were isolated from cortices of E/Nestin:EGFP transgenic mice at postnatal day 7 as previously described [28]. Briefly, cortices were dissected out after removing cerebellum, olfactory bulb, meninges, and peripheral blood vessels. After washed twice with HBSS, cortices were digested at 37 °C for 20 min in 0.25% trypsin solution supplemented with 0.05% DNase I. Digestion was stopped by FBS (Invitrogen). The tissue sediment was centrifuged at 375 g for 5 min at room temperature (RT) and washed twice with HBSS. After trituration, cells were cultured in DMEM/F12 with 10% FBS, 50 U penicillin and 50 mg/mL streptomycin at 37 °C. The culture medium was replaced every 3 days. Cells were subjected to three passages for purification purpose. Astrocyte purity was tested by immunostaining with antibodies against GFAP (DAKO) and GLAST (Sigma).

### Reagents

For adherent NPC cultures, tissue culture dishes or plates were coated with 100 μg/mL Poly-D-Lysine (Sigma) and 5 μg/mL Fibronectin (Sigma). Mouse AiNPCs and control NPCs were cultured in NPC favoring medium (NPCM), which contains NeuroCult® NSC Basal Medium (Stem Cell Technologies), NeuroCult® NSC Proliferation Supplements (Stem Cell Technologies), 20 ng/mL bFGF (BioWalkersville), 20 ng/mL EGF (BioWalkersville) and 2 μg/mL heparin (Sigma). AiNPCs and control NPCs were passaged with TrypLE™ select (Invitrogen). Plat-E packaging cells were cultured in DMEM with high glucose (Gibco) containing 10% heat inactivated FBS, 50 U penicillin and 50 mg/mL streptomycin, 1 μg/mL puromycin (Sigma) and 10 μg/mL blasticidin S (InvivoGen).

Control NPCs used in this study were generated from E15.5 mouse cortices. Briefly, the cortices were dissociated into single cells by triturating the tissue sediments with a 1 mL pipette. Cells were seeded into 100 mm non-coated Petri dishes (Fisher) at a density of 2 × 10^5^ cells/mL in 10 mL of NPCM for primary neurosphere formation. Culture medium was replaced every other day. Neurospheres were passaged every 4–6 days when they reached 150 μm in diameter.

iPSC derived from mouse cortical astrocytes and fibroblasts were generated and characterized in our lab as previously described [17].

### Retroviral vectors and retrovirus preparation

Plasmid encoding mouse Sox2 was purchased from Addgene (Plasmid #13367). Mouse Foxg1 (restriction enzymes: BamHI and XhoI), Brn2 (restriction enzymes: BamHI and XhoI), Lhx8 (restriction enzymes: BamHI and SalI), Foxa2 (restriction enzymes: Xho1 and Sal1) and Lmx1a (restriction enzymes: BamH1 and SalI) were amplified from mouse control NPCs cDNA library. Each gene was individually cloned into pMXs-retroviral vectors (Cell Biolabs, RTV-010).

Retroviruses (pMXs) were generated with Plat-E packaging cells as previously described [11]. Constructed pMXs-based retroviral plasmids along with empty pMXs-vector control were introduced into Plat-E cells using Lipofectamine® LTX with Plus™ transfection reagent (Invitrogen) according to the protocol from the manufacturer. 24 h after transduction, medium was changed to astrocyte favoring medium for astrocyte transduction or NPCM for AiNPC transduction. 48 h after transfection, virus containing supernatants were filtered with 0.45 μm cellulose acetate filters (Fisher) and immediately used for astrocyte reprogramming.

### Reprogramming of mouse astrocytes

NPC direct reprogramming was performed with an adopted and modified protocol as previously described [3]. Briefly, mouse astrocytes were incubated in the mixed virus-containing supernatants overnight. 10 μg/mL polybrene (Millipore) was added during the viral vector infection to increase the transduction efficiencies. A second round of infection was performed using the same mixed virus-containing supernatants. Infected astrocytes were changed to NPCM culture 1 day after the second infection. NPCM was replaced every 2 days. A drastic cell morphology change was observed from day 4 post infection. Patches of highly proliferating transduced cells formed dense networks and by day 28 bulging Nestin-EGFP^+^ colonies were found aggregated at the intersections of cell patches. On day 30 after retroviral transduction, bulged Nestin-EGFP^+^ colonies were manually picked, pooled, and suspended into single cells in Petri dishes. After culturing for 4–6 days, floating primary neurospheres were collected and re-plated into Poly-D-Lysine/Fibronectin coated 6 well plates. Upon 80% confluency, cells were collected and resuspended into single cells for a second round neurosphere formation in suspension culture. After 3 rounds of selection and enrichment, cells were passaged either in adherent culture or suspension culture according to the requirements of further experiments.

### NPC Neurosphere formation assay

NPC neurosphere formation (self-renewal) assay was performed by suspending 1.0 × 10^4^ cells with NPCM in a 60-mm non-coated Petri dish. Fresh medium was added into the suspension culture every other day. On day 5 neurospheres were collected and the number of neurospheres was counted under the bright field of a microscope.

### Immunocytochemistry

Cells were fixed in 4% paraformaldehyde (Sigma) for 15 min at RT, rinsed 3 times with PBS (Fisher), and then incubated with permeabilizing/blocking buffer containing 5% goat serum (Vector Laboratories) and 0.2% Triton X-100 (Bio-Rad) in PBS for 60 min at RT. Primary antibodies were added overnight at 4 °C. The following day cells were washed 3 times with PBS and incubated with secondary antibodies (Molecular Probes) for 60 min at RT. Cells were counterstained with DAPI (Sigma-Aldrich). IgG control was used as negative controls for all immunocytochemical analysis. Images were captured using a Nikon Eclipse E800 microscope equipped with a digital imaging system and imported into Image-ProPlus, version 7.0 (Media Cybernetics, Sliver Spring, MD) for quantification. Images were imported into Image-Pro Plus, version 7.0 (Media Cybernetics, Silver Spring, MD), for quantification and 600–1,000 immunostained cells from 15 random fields per group were counted.

### RNA isolation and qPCR analysis

Total RNA was isolated by RNA Purification Kit (Fermentas), and DNase I digestion was included (Qiagen) to remove genomic DNA. mRNA derived cDNA was generated through Oligo-dT priming with Transcriptor First Strand cDNA Synthesis Kit (Roche). RNase inhibitor was used to prevent RNA degradation during reverse transcription. Amplification was performed with SYBR Green PCR Master Mix (Applied Biosystems) and specific primer sets (Additional file 1: Table S2). All mRNA expression levels were normalized to GAPDH and calibrated on the control cells specified in each experiment.

### Neuronal differentiation

Basic neuronal differentiation medium contained DMEM/F12 (without HEPEs), 1x N2 (Invitrogen), 1x B27 (Invitrogen), 1 μM cAMP (Sigma-Aldrich) and 0.2 mM Ascorbic Acid (Sigma-Aldrich). Spontaneous neuronal differentiation was carried out on Poly-D-Lysine/Fibronectin coated coverslip as previously described [29]. The single-cell spontaneous differentiation assay was performed by plating 10^4^ cells/well on 24-well plates in basic neuronal differentiation medium, 10 ng/mL BDNF (R&D Systems), 10 ng/mL GDNF (PeproTech), 10 ng/mL IGF (PeproTech), 10 ng/mL CNTF (ProSpec) for 14 to 28 days. Medium was changed every 3 days. For ventral mesencephalic “inducing” differentiation, cells were treated with DMEM/F12 (without HEPEs), 1x N2, 1x B27, 100 ng/mL murine N-terminal fragment of SHH (R&D Systems) and murine 100 ng/mL FGF8 isoform b (R&D Systems) for 6 days followed by 14 days in DMEM/F12 (without HEPEs), 1 mM cAMP (Sigma) and 200 mM AA (Sigma). Medium was changed every 3 days.

### Pyrosequencing and DNA methylation analysis

Genomic DNA was isolated using DNeasy Blood & Tissue Kit (Qiagen). Bisulfite treatment was carried out using 1000 ng of DNA and the EZ DNA Methylation-Direct kit (Zymo Research). This process deaminated unmethylated cytosine (C) residues to uracil (U) leaving methylated cytosine (^m^C) residues unchanged. To perform PCR reactions, 32 ng of bisulfite-treated DNA was used as template. The PCR reactions were performed in a total volume of 25 μl for 35 cycles using 1.0 U FastStart Taq DNA Polymerase (Roche), 3.5 mM MgCl2 solution, 0.2 mM dNTPs, 0.24 μM sense primer, 0.18 μM antisense primer (Additional file 1: Table S3) under the following conditions: 95 °C for 30 s, 45 s at annealing temperature (Additional file 1: Table S3) and 72 °C for 1 min. Human lymphocyte genomic DNA (Roche) was used as a positive control (high methylation level) and was methylated using M. SssI (CpG) methylase kit (New England Biolabs) followed by sodium bisulfite treatment as described above. The negative control (low methylation level) was obtained by treating 1000 ng human lymphocyte genomic DNA with sodium bisulfite directly. All PCR products were electrophoresed and visualized under a Bio-Rad Laboratories Gel-Doc UV illuminator (Hercules). Methylation percentage of each CpG was determined using a Qiagen Pyromark Q24 pyrosequencer (Valencia) and sequencing primer (Additional file 1: Table S3), according to recommendations from manufacturer.

### Statistical analyses

Data were evaluated statistically by the analysis of variance (ANOVA), followed by a Tukey’s test for multiple comparisons (Graphpad Prism 5.0 software). Data were shown as mean ± SD, and significance was determined as *P* < 0.05. Means between two groups were compared with two-tailed, paired or unpaired Student’s t tests.

## Results

### Conversion of mouse astrocytes into iNPCs

**Fig. 1:**
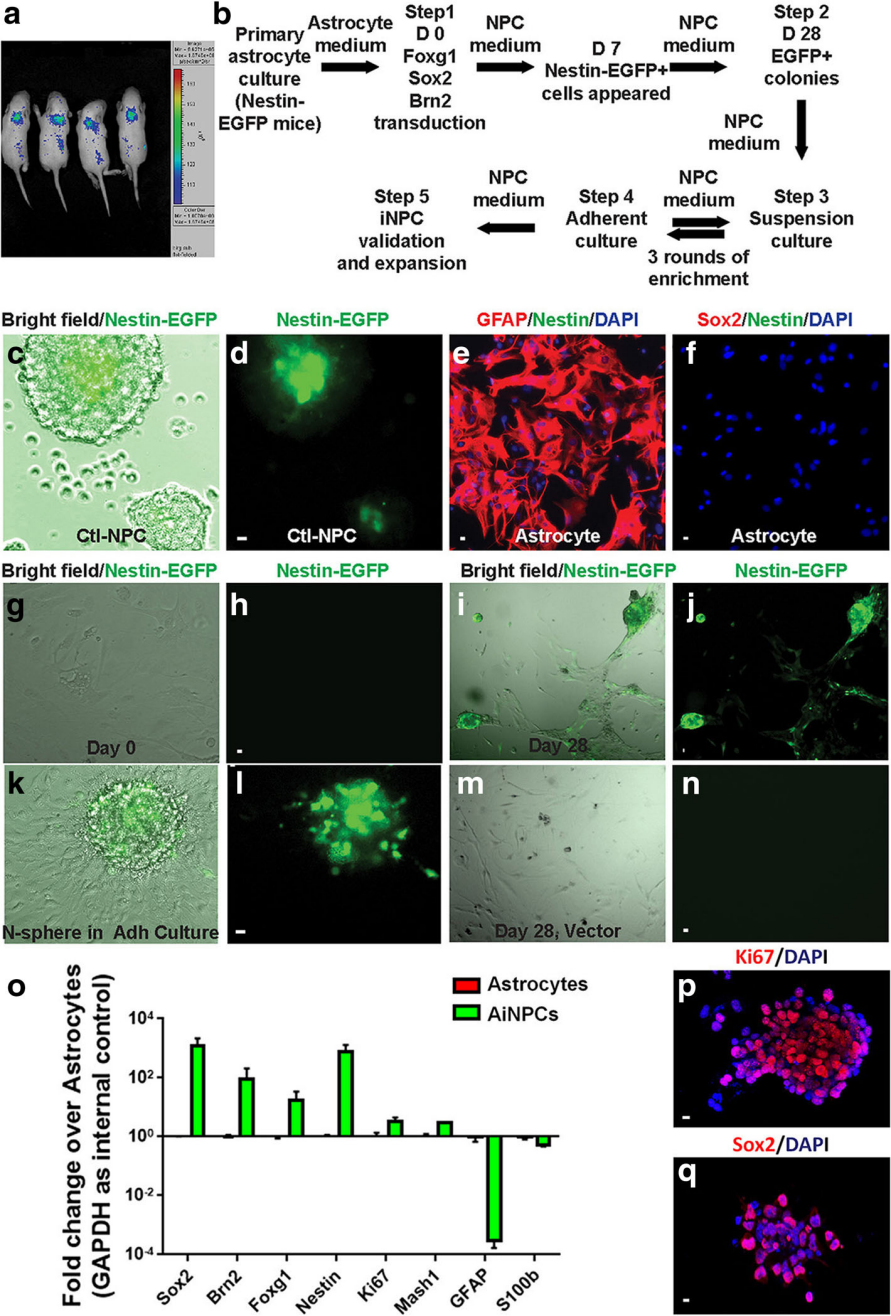
Reprogramming of mouse astrocytes into AiNPCs. **a** Nestin:EGFP transgenic mice were imaged under IVIS optical imaging system. **b** Schematics for the reprogramming of astrocytes into AiNPCs. **c**, **d** Nestin-EGFP^+^ neurospheres were derived from E15.5 Nestin:EGFP transgenic mouse cortices in suspension culture. **e**, **f** Primary astrocyte cultures were positive for GFAP, but negative for Sox2 and Nestin. **g**-**n** Representative bright field and fluorescent pictures of cultures during generation of GFP^+^ NPC-like cells were shown. Step 1, day 0, astrocytes were in adherent culture before retrovirus-mediated Foxg1 + Sox2 + Brn2 transduction (**g**, **h**). Step 2, day 28, the cultures formed multiple bulged Nestin-EGFP^+^ colonies (**i**, **j**). Step 3 & 4, Nestin-EGFP^+^ primary neurospheres appeared and NPC-like cells migrated from neurospheres during adherent culture in poly-D-lysine- and fibronectin-coated dishes (**k**, **l**). Mock treated astrocytes showed minimum levels of Nestin-EGFP (**m**, **n**). **o** RNA of astrocytes and AiNPCs was collected and expression of NPC- and glial-specific genes was analyzed using qPCR. Data were normalized to GAPDH and presented as fold change compared with astrocytes. **p**, **q** AiNPCs were positive for Ki67 and Sox2. Scale bars represent 10 μm (**c**-**n**, **p**, **q**). Error bars denote s.d. from triplicate measurements (**o**)

To facilitate the observation of the somatic reprogramming toward a neural progenitor fate, we used Nestin:EGFP transgenic mice, where Nestin positive cells expressed enhanced GFP fluorescence. Under the IVIS optical imaging system, Nestin:EGFP positive transgenic mice at postnatal day 7 showed strong EGFP signals in the brain (Fig. 1a). To reprogram EGFP negative astrocytes into EGFP positive iNPCs, we employed a set of TFs (Foxg1, Sox2, and Brn2) that were previously used in the reprogramming of fibroblast into iNPC (Fig. 1b) [19]. Control NPCs, which formed floating neurosphere, were derived from cortices of embryonic day 15.5 Nestin:EGFP transgenic mice. These NPCs served as positive controls for the fibroblast-derived iNPCs. Green fluorescence of Nestin:EGFP was readily detectable from those control NPC neurospheres under fluorescent microscope, indicating the expression of Nestin, one of NPC-specific marker genes (Fig. 1c, d). For reprogramming, astrocytes were derived from cortices of Nestin:EGFP transgenic mice at postnatal day 7. Over 95% of cells in the primary culture were positive for glial fibrillary acidic protein (GFAP) and no Nestin, Sox2, Iba1 and Tuj1 staining was detected in the astrocyte cultures, suggesting the purity of astrocyte culture in the absence of NPCs, microglia and neurons (Fig. 1e, f; Additional file 1: Figure S1). This enriched astrocyte culture was negative for any EGFP signal and was used as the starting cells for somatic reprogramming (Fig. 1g, h). To reprogram astrocytes into NPCs, we transduced them with mixed retroviruses that individually expressed either Sox2, Brn2, or Foxg1 and subsequently changed the medium to NPCM. At 7 days after transduction, Nestin^+^ cells emerged, which could be identified through EGFP expression. These EGFP^+^ cells proliferated and formed neurospheres in the cultures. At 28 days after transduction, EGFP positive colonies were collected, dissociated into single cells, and sub-cultured in suspension as neurospheres (Fig. 1i, j). These neurospheres maintained strong EGFP fluorescence and could be continuously sub-cultured (Fig. 1k, l). Postnatal cultured astrocytes are known to form spherical clones in the presence of EGF and bFGF (Laywell et al., PNAS 2000). To rule out that the NPCs obtained from the transduced astrocyte cultures are not from Sox2, Brn2, and Foxg1 transduction but from spontaneously formed spherical clones, we used astrocytes transduced with the same titer of the retroviral vector and subsequently cultured with the same NPCM as the control cultures. No EGFP signal or neurospheres was observed in the control group (Fig. 1m, n) [30]. Therefore, we concluded that the NPCs obtained from the transduced astrocyte cultures were the result of Sox2, Brn2, and Foxg1 transduction. The transduction efficiency of Sox2, Brn2, and Foxg1 was validated by qPCR. Compared with those in the starting astrocytes, the overall gene expression levels of Sox2, Brn2, and Foxg1, which included both endogenous (from normal gene transcription) and exogenous (from retroviral vectors) in transduced astrocytes or reprogrammed NPCs, which we referred to as AiNPCs, were upregulated. Similarly, the NPC-specific gene nestin, neural lineage gene Mash1, and proliferation marker gene Ki67, were all upregulated. As expected, astrocyte-specific genes, including GFAP and S100b, were downregulated compared with those in the starting (Fig. 1o). To differentiate between endogenous and exogenous gene transcripts of Sox2, Brn2, and Foxg1, we designed primers specific for each transcript. Endogenous gene expression levels of Sox2, Brn2, and Foxg1 in transduced astrocytes or AiNPCs, were downregulated compared with those in the control group. In contrast, exogenous gene expression of Sox2, Brn2, and Foxg1 from the retroviral vectors in transduced astrocytes or AiNPCs were significantly upregulated compared with those in the control group, suggesting that the elevation of Sox2, Brn2, and Foxg1 transcript levels was mainly due to the exogenous expression of these genes (Additional file 1: Figure S2). Consistent with high mRNA levels of Sox2 and Ki67, AiNPCs showed strong immunoreactivities to Sox2 and Ki67, suggesting a very active proliferative status of AiNPCs (Fig. 1p, q).

### Characterization of AiNPC

**Fig. 2:**
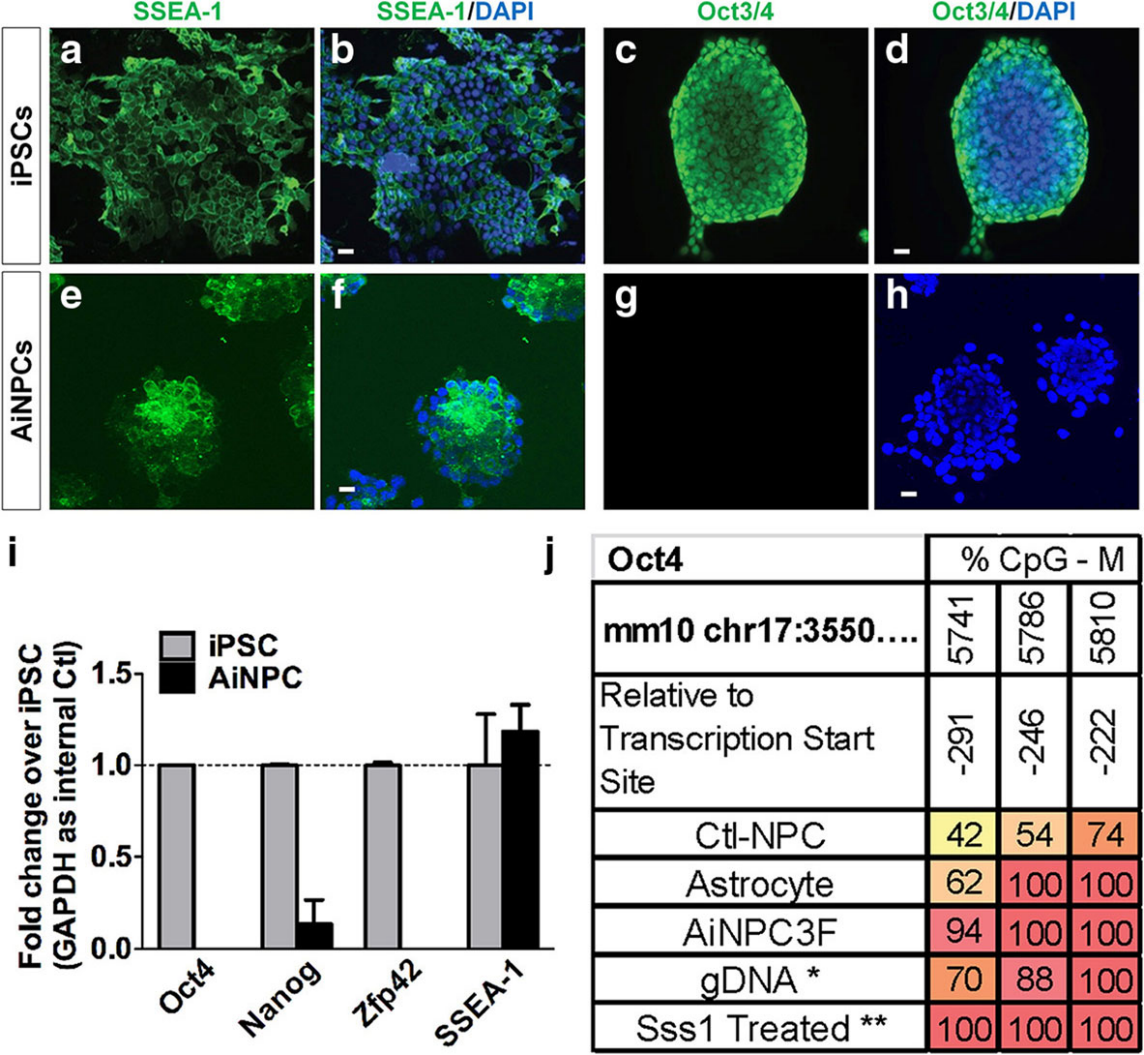
Absence of pluripotency markers in AiNPCs. **a**-**h** iPSC and AiNPC cultures were fixed and immunostained for pluripotency markers SSEA-1 and Oct4. **i** RNA of cultured AiNPCs and control iPSCs was collected and expression of pluripotent-associated genes was analyzed using qPCR. Data were normalized to GAPDH and presented as fold change compared with control iPSCs. Error bars denote s.d. from triplicate measurements. **j** The Oct4 promoter regulatory region DNA methylation patterns of AiNPCs, control astrocytes, and control NPCs were analyzed using pyrosequencing method. *Human lymphocyte genomic DNA was used as a negative control for pyrosequencing. **Sss1 methyltranferase treated human lymphocyte genomic DNA was used for positive control. Scale bars represent 10 μm (**a**-**h**). Error bars denote s.d. from triplicate measurements (**i**)

Next, we characterized the AiNPCs with multiple approaches. First, we examined whether the AiNPCs manifested any phenotypes of pluripotency. SSEA-1, a marker shared by embryonic stem cells (ESCs), induced pluripotent stem cells (iPSCs) and NPCs/iNPCs [13, 31], was expressed in AiNPCs at comparable levels to iPSCs (Fig. 2a, b, e, f). In contrast, Oct4, a pluripotency marker specific for iPSCs (Fig. 2c, d), was absent in AiNPCs (Fig. 2g, h). Consistent with the immunostaining data, AiNPC expressed comparable levels of SSEA-1 gene transcripts but expressed significantly lower levels of pluripotency mark genes Oct4 compared to iPSCs (Fig. 2i). Furthermore, other pluripotency markers Nanog and Zfp42 [3] were also expressed at significantly lower levels in AiNPCs compared to iPSCs (Fig. 2i). Pyrosequencing on bisulfite-treated DNA showed the regulatory region on the promoter region of Oct4 [32], but not that of SSEA-1 was hypermethylated, confirming the transcriptional silencing of Oct4 (Fig. 2j, Additional file 1: Figure S3). Together, these data suggest that the AiNPCs likely do not go through the stage of the pluripotency.

**Fig. 3:**
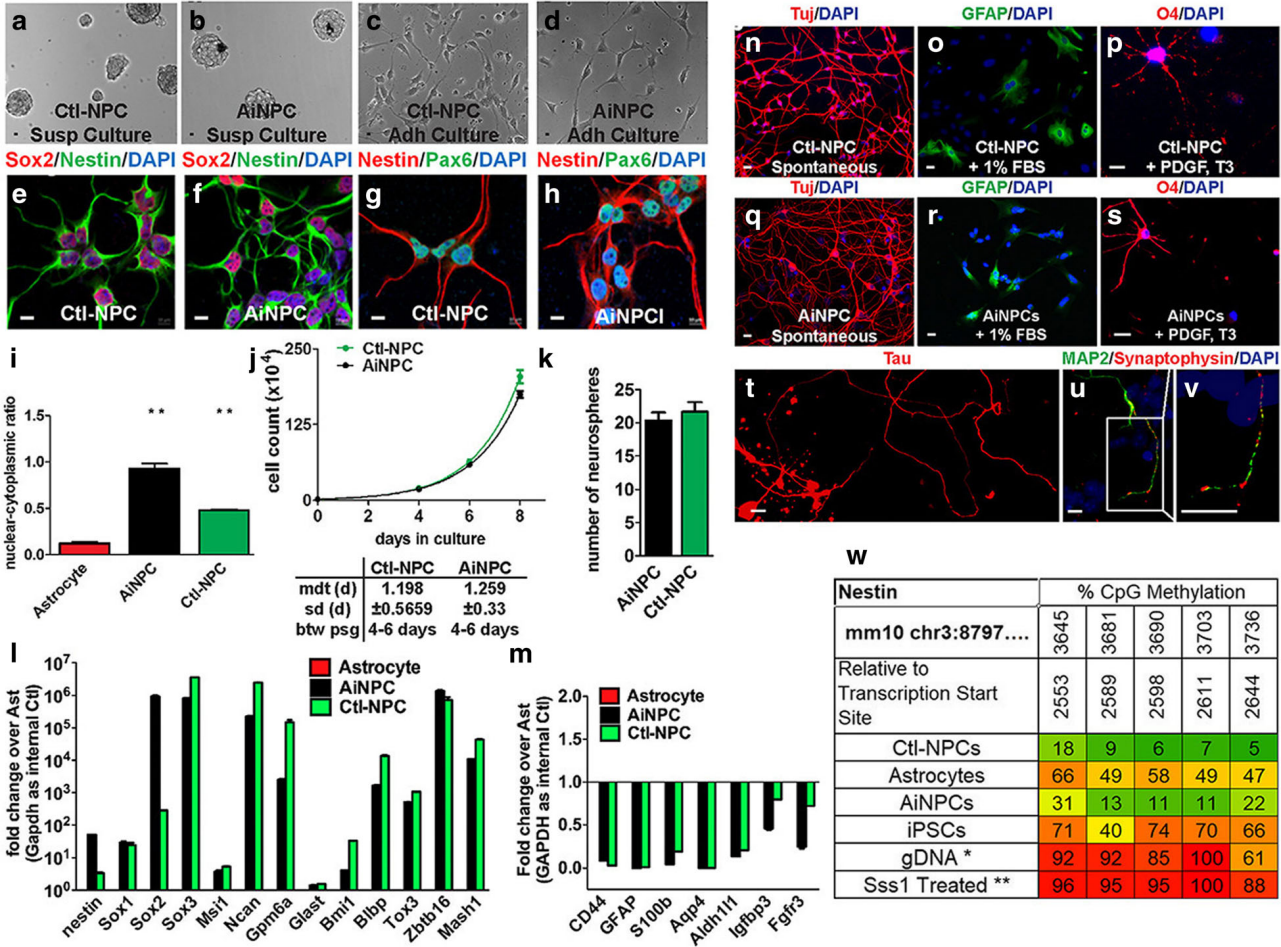
Validation and characterization of AiNPCs. **a**-**d** Bright-field of AiNPCs showed a typical NPC morphology in both suspension and adherent cultures, compared to control NPCs. **e**-**h** AiNPCs expressed NPC markers Nestin, Sox2, and Pax6. **i** Nuclear-cytoplasmic ratios of AiNPCs, control astrocytes, and control NPCs were assessed by quantifying the size of nucleus and cytoplasm in Image-Pro Plus. More than 100 cells per groups were randomly chosen for each measurement. **P < 0.01 by two-tailed t test (*n* = 3). **j** Proliferation of AiNPCs and control NPCs were determined by counting the total cell number during each passage. Mdt, mean doubling time. Sd, standard deviation. Btw psg, between passages. **k** Total neurosphere number per 100 AiNPCs or control NPCs were counted to determine the neurosphere forming efficiencies. **l**, **m** RNA of AiNPCs, control astrocytes, and control NPCs were collected and the expression of astrocytic differentiation marker genes (**l**) and NPC marker genes (**m**) was analyzed by qPCR. Data were normalized to GAPDH and presented as fold change compared with control astrocytes. **n**-**v** Control NPCs and AiNPCs were placed in neuronal (**n**, **q**, **t u**, **v**), astrocyte (**o**, **r**), and oligodendrocyte (**p**, **s**) differentiation media. Cultures were fixed and stained with Tuj1 (**n**, **q**), GFAP (**o**, **r**), O4 (**p**, **s**), Tau (**t**), and MAP2/synaptophysin (**u**, **v**). (**w**) The Nestin promoter regulatory region DNA methylation patterns of AiNPCs, control astrocytes, and control NPCs were analyzed using a pyrosequencing method. *Human lymphocyte genomic DNA was used as a negative control for pyrosequencing. **Sss1 methyltranferase treated human lymphocyte genomic DNA was used for positive control. Scale bars represent 10 μm (**a**-**h**, **n**-**v**). Error bars denote s.d. from triplicate measurements (**i**, **k**, **l**, **m**)

Second, we tested whether AiNPCs shared key characteristics with control NPCs that generated from E15.5 mouse brains. Morphologically, AiNPCs highly resembled control NPCs isolated and cultured from E15.5 mice cortices in both suspension and adherent culture (Fig. 3a-d). Furthermore, AiNPCs were positive for NPC markers such as Nestin, Pax6, and Sox2, similar to control NPCs, suggesting that the AiNPCs possessed molecular characteristics of NPCs (Fig. 3e-h). In addition, the AiNPCs were similar in both nuclear-cytoplasmic ratio and efficiency in proliferative and self-renewal capacities with control NPCs (Fig. 3i-k). qPCR further revealed that AiNPCs expressed all endogenous NPC marker genes that we detected at levels close to the control NPCs, including Nestin, Sox1, Sox2, Sox3, Msi1, Ncan, Gpm6a, Bmi1, Blbp, Tox3, and Zbtb16 (Fig. 3l). In contrast, astrocytic marker gene expressions were suppressed after reprogramming (Fig. 3m). The DNA methylation analysis of the promoter of Nestin revealed low methylation status in AiNPCs, confirming the activation of the endogenous Nestin gene (Fig. 3w). Finally, we looked at the multipotency of AiNPCs and found that similar to NPCs (Fig. 3n-v), AiNPCs spontaneously differentiated into Tuj1^+^ neurons, GFAP^+^ astrocytes, and O4^+^ oligodendrocytes after culturing in cell type-specific differentiation media for 14 days (Fig. 3n-s). In the prolonged differentiation cultures (3 weeks), positive labeling of MAP-2, Tau, and Synaptophysin was found, suggesting the presence of axons and neuronal presynaptic structures that are hallmarks of mature neurons (Fig. 3t-v). Together, these extensive characterizations strongly support the NPC status of the AiNPCs.

### The telencephalic-phenotype and neuronal subtype specification of AiNPCs

**Fig. 4:**
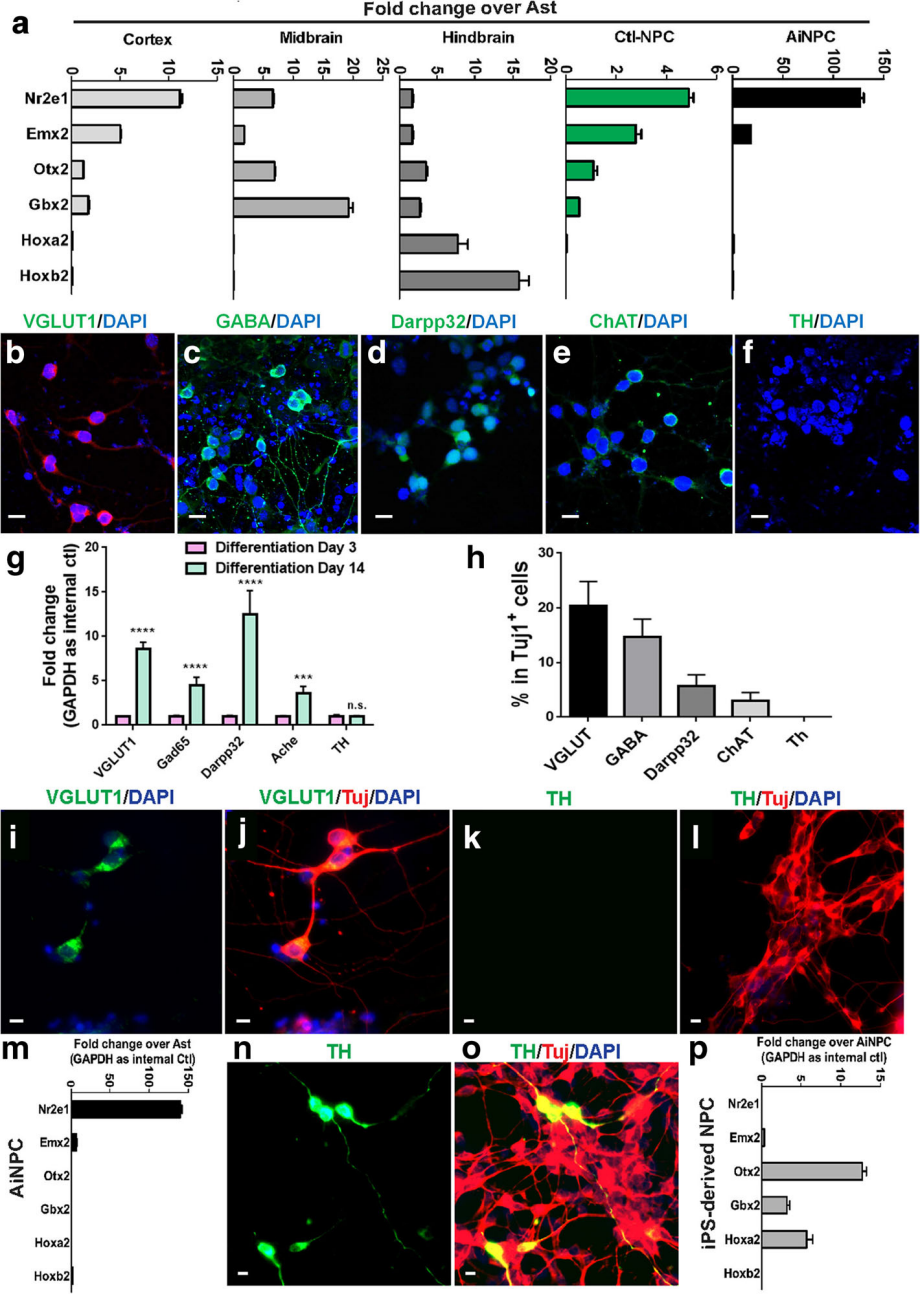
Telencephalic-like regional identity and neuronal subtype specification of AiNPCs. **a** Cortex, midbrain, hindbrain, cultured NPCs, and AiNPCs regional-specific gene expression pattern was determined by qPCR. **b**-**e** AiNPCs were placed in specific neuronal subtype differentiation media, VGLUT1^+^ glutamatergic neurons (**b**), GABA^+^ inhibitory neurons (**c**), Darpp32^+^ inhibitory neurons (**d**), ChAT^+^ cholinergic neurons (**e**), and TH^+^ dopaminergic neurons (**f**) were identified through immunocytochemistry. **g** Expression of maker genes in AiNPC-derived glutamatergic (VGLUT1), GABAergic (Gad65, Darpp32), cholinergic (Ache) and dopaminergic (Th) neurons was determined by qPCR. **h** Proportions of neuronal subtypes generated from AiNPCs were determined by immunocytochemistry and shown as a percentage to total neuronal numbers. **i**-**m** AiNPCs were placed under mesencephalic cue and glutamatergic/dopaminergic neuron-specific markers and regional-specific gene expressions were determined by immunocytochemistry (**i**-**l**) and qPCR analysis (**m**), respectively. **n**-**p** Under the same mesencephalic cue, a dopaminergic neuron-specific marker and regional-specific gene expressions were determined by immunocytochemistry (**n**, **o**) and qPCR analysis (**p**), respectively. Scale bars represent 10 μm (**b**-**f**, **i**-**l**, **n**, **o**). Error bars denote s.d. from triplicate measurements (**a**, **g**, **h**, **m**, **p**)

In the developing brain, gene expression pattern changes following a spatiotemporal order based on their embryonic anatomical locations [33, 34]. The gene expression pattern, collectively depicting the regional identity of NPCs, is known to closely associate with specific neuronal lineages [35], so we examined the region-specific gene expression patterns of the AiNPCs by qPCR. The expression levels of marker genes along the anterior-posterior axis confirmed the regional identities of the tissues isolated from the cortex, midbrain, or hindbrain (Fig. 4a). Based on the marker genes expression, control NPCs had a distinct cortical identity. Similarly, AiNPCs expressed markers of cortex but not that of midbrain or hindbrain. Telencephalic NPCs from midgestation mouse embryos (E10.5) were lineage-restricted and refractory to ventral mesencephalic cues for inducing dopaminergic neurons [29].

Next, we investigated the neuronal subtypes that could be derived from the AiNPCs. Interestingly, under spontaneous neuronal differentiation condition, AiNPCs were able to differentiate into VGLUT^+^ glutamatergic neurons, GABA^+^/Darpp32^+^ inhibitory neurons, and ChAT^+^ cholinergic neurons, but not TH^+^ dopaminergic neurons (Fig. 4b-f). This specific pattern of neuronal subtype differentiation is supported by qPCR analysis of gene transcripts. The transcription levels of VGLUT1 (glutamatergic neuron), Gad65 and Darpp32 (GABAergic neuron), and Ache (cholinergic neuron) were at significantly higher levels in AiNPCs-differentiated cells collected at 14 days after differentiation, compared to AiNPCs-differentiated cells collected at 3 days after differentiation. In contrast, the transcription levels of TH (dopaminergic neuron marker) remained unchanged during differentiation (Fig. 4g). Quantification of Tuj1^+^ cells expressing neuronal subtype-specific markers suggested that the differentiation of AiNPCs was predominantly toward the glutamatergic and GABAergic neurons, occasionally toward cholinergic neurons, but rarely toward dopaminergic neurons (Fig. 4h).

To test whether AiNPCs still lack dopaminergic differentiation capacity when put in a ventral mesencephalic “inducing” condition, we adapted a differentiation protocol that involved SHH and FGF8 [21, 29, 36] and found that in such condition the gene expression of AiNPCs still represented a distinct telencephalic pattern (Fig. 4i-m). The mouse astrocyte-derived iPSCs and iPSCs-derived NPCs were used as positive controls [37]. Unlike AiNPCs, the expression of regional markers associated with midbrain/rostral hindbrain and dopaminergic markers could be induced in NPCs derived from iPSCs in the same culture condition (Fig. 4n-p). This restricted neuronal subtype specification suggested that AiNPCs had committed to a telencephalic lineage that predominantly generated glutamatergic and GABAergic neurons.

### The induction of cholinergic neuron differentiation by additional Lhx8 overexpression

**Fig. 5:**
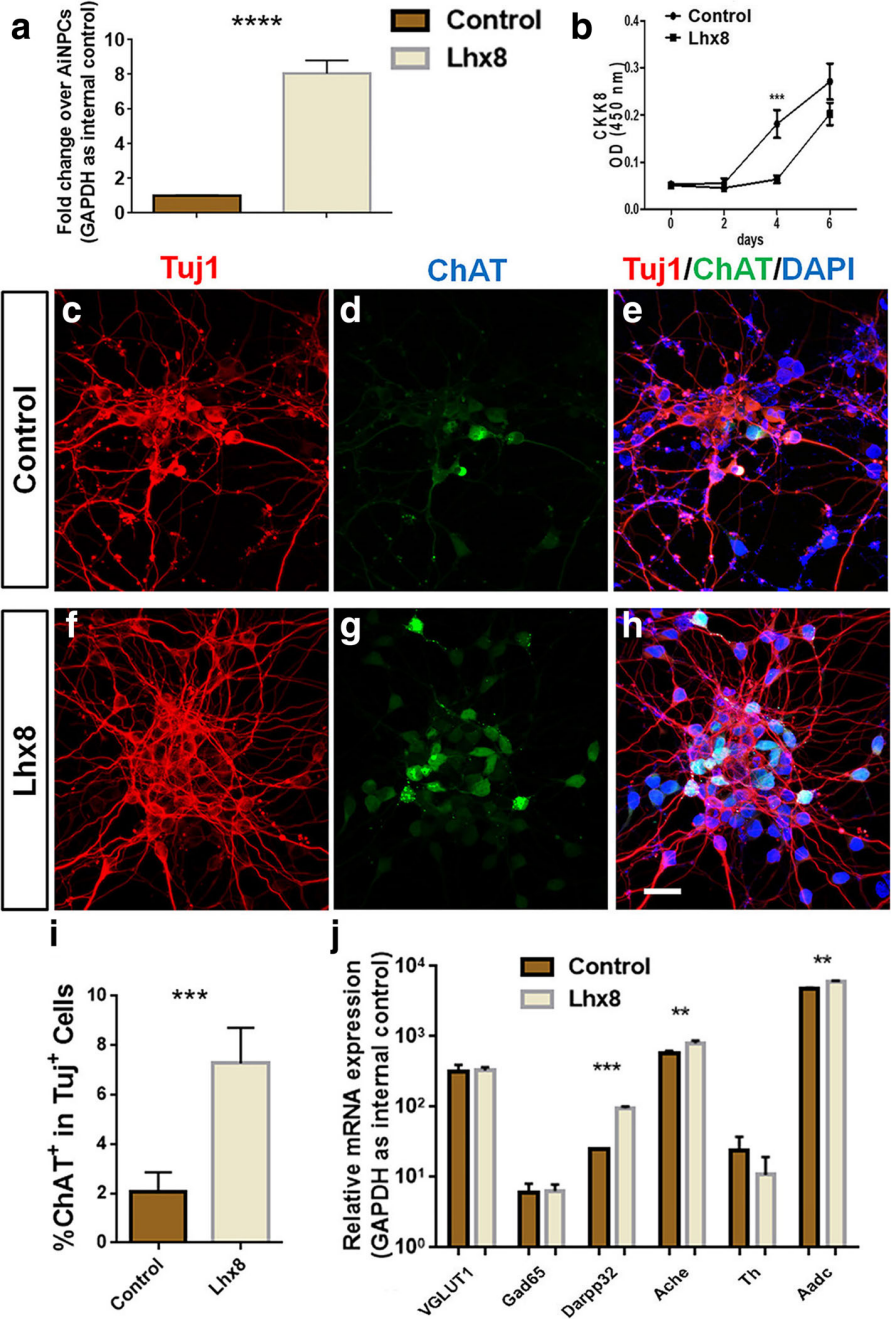
Forced expression of Lhx8 in AiNPCs enhances cholinergic neuron generation. **a** Following overexpression of Lhx8, the level of Lhx8 in AiNPCs was determined by qPCR. **b** The proliferation of AiNPCs after overexpression of Lhx8 was determined by CCK-8 assay. **c**-**h** Augmentation of ChAT^+^/Tuj1^+^ cholinergic neuron generation after transduction of Lhx8 in AiNPCs were determined by immunocytochemistry. **i** The percentage of ChAT^+^/Tuj1^+^ neurons in panels c-h was quantified by counting ChAT^+^ cells and comparing against the total number of Tuj^+^ neurons. ***P < 0.001 by two-tailed *t* test (*n* = 3). **j** Neuronal subtype marker gene expressions in Lhx8-transduced AiNPCs were determined by qPCR. **P < 0.01, ***P < 0.001 by two-tailed *t* test (*n* = 3). Scale bars represent 20 μm (**c**-**h**). Error bars denote s.d. from triplicate measurements (**a**, **b**, **i**, **j**)

Emerging evidence demonstrated that the loss of cholinergic neurons is directly linked with the pathogenesis of Alzheimer’s disease (AD) [38, 39]. Inefficiency of cholinergic neuronal differentiation from iNPCs has become a critical roadblock for cell transplantation based therapeutic strategy of AD. Recent studies suggested that Lhx8 is a key facilitator of cholinergic neurogenesis, therefore, we hypothesized that Lhx8 involves in the cell fate commitment of AiNPCs towards cholinergic lineage. To test the hypothesis, we first overexpressed Lhx8 in AiNPCs through the pMXs-retroviral vector. The overexpression of Lhx8 was confirmed in AiNPCs by qPCR at 4 days post transduction (Fig. 5a). After Lhx8 overexpression, no difference in proliferation as indicated by the CCK-8 analysis, was observed in AiNPCs except at the day 4, suggesting that Lhx8 has no effect on proliferation of AiNPCs (Fig. 5b). Importantly, through immunocytochemical analysis, we found that the percentage of ChAT^+^ cholinergic neurons generated from AiNPCs was significantly increased following the overexpression of Lhx8 (Fig. 5c-i). In contrast, Lhx8 overexpression had no significant impact on the generation of other neuronal subtypes in our immunocytochemical analysis (Additional file 1: Figure S4). These immunocytochemical data were corroborated by qPCR that demonstrated significant increases of gene transcripts corresponding to the marker of cholinergic neurons (Ache) but not those of glutamatergic neurons (VGLUT1), GABAergic neurons (Gad65), and dopaminergic neurons (Th) (Fig. 5j). Together, these data suggested that Lhx8 specifically promotes the generation of cholinergic neurons from AiNPCs.

### The induction of dopaminergic neuron differentiation by additional Foxa2 overexpression

**Fig. 6:**
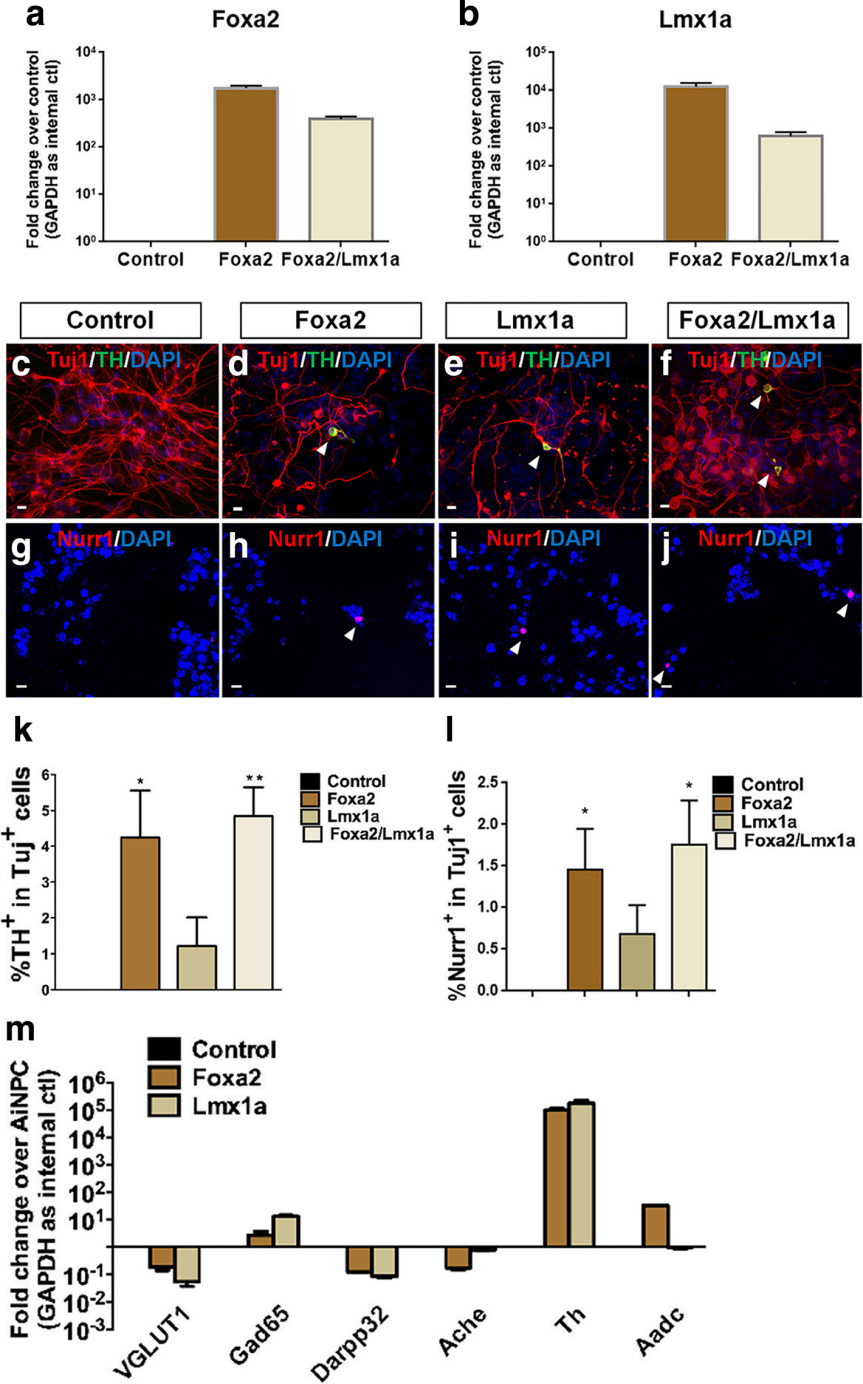
Forced expression of Foxa2/Lmx1a in AiNPCs enhances dopaminergic neuron generation. **a**, **b** Following overexpression of Foxa2/Lmx1a, the levels of Foxa2 and Lmx1a in AiNPCs were determined by qPCR. **c**-**j** Augmentation of TH^+^/Nurr1^+^ dopaminergic neuron generation after transduction of Foxa2 (**d**, **h**), Lmx1a (**e**, **i**), and Foxa2/Lmx1a (**f**, **j**) in AiNPCs were determined by immunocytochemistry. **k**, **l** The percentage of TH^+^/Nurr1^+^ neurons in panels c-j was quantified by counting TH^+^/Nurr1^+^ cells and comparing against the total number of Tuj^+^ neurons. *P < 0.05, **P < 0.01 by two-tailed *t* test (*n* = 3). **m** Neuronal subtype marker gene expressions in Foxa2- and Lmx1a-transduced AiNPCs under mesencephalic cues were determined by qPCR. Scale bars represent 10 μm (**c**-**j**). Error bars denote s.d. from triplicate measurements (**a**, **b**, **k**-**m**)

Degeneration of midbrain dopaminergic neurons is a key pathological event of Parkinson’s disease (PD). The cell-based therapy for PD requires high efficiency of dopaminergic neuron generation. To determine whether midbrain dopaminergic fate could be achieved from the forebrain-specific AiNPCs, we further included two more TFs, Foxa2 and Lmx1a, that are crucial in midbrain dopaminergic neuron differentiation [23, 40, 41]. We used pMX retroviral gene delivery system to transduce Foxa2 and Lmx1a into AiNPCs. The overexpression of Foxa2 and Lmx1a was evident in AiNPCs at 4 days post transduction (Fig. 6a, b). After cultured under ventral mesencephalic cue SHH and FGF8 for 1 week, AiNPCs acquired dopaminergic neurogenesis capability after the transduction of individual Foxa2, Lmx1a, or Foxa2/Lmx1a in combination (Fig. 6c-j). Interestingly, TH^+^/Nurr1^+^ dopaminergic neuron yields in Foxa2- and Foxa2/Lmx1a-transduced AiNPCs were significantly higher than Lmx1a-transduced AiNPCs (6 k, l), suggesting that Foxa2 is a stronger factor for dopaminergic neuronal induction compared with Lmx1a. qPCR analysis of the differentiated AiNPCs confirmed the predominant induction of dopaminergic genes (Th and Aadc) but not that of glutamatergic, GABAergic, and cholinergic genes (Fig. 6m).

## Discussion

Adult brain is known to have limited regeneration after injury. During neurodegenerative diseases, the limited regeneration is often not sufficient to compensate for the loss of neuronal functions [4, 42]. The reprogramming of somatic cells to replace the damaged neurons is a promising therapeutic strategy in treating neurodegenerative diseases [43, 44]. Recently, astrocyte-based reprogramming has received growing interest within the scientific community due to its abundance and regenerative capacity [21, 45–49]. Two main approaches are typically applied in these studies. One approach is to directly convert astrocytes into neuronal cells [45–47]. This approach may be more specific and less tumorigenic. However, limitations in reprogramming efficiency and cell number curb broad functional recoveries in the brain. Another approach is to reprogram astrocytes into proliferative iNPCs [21, 49]. This approach could overcome the cell number limitation and is applied in the current study. Using retroviral vectors that overexpressed TFs Foxg1, Sox2, and Brn2, we successfully reprogramed mouse astrocytes into iNPCs without going through the stage of iPSCs. The AiNPCs exhibited typical NPCs’ phenotype, including the self-renewal and the tripotency to differentiate into neurons, astrocytes, and oligodendrocytes under defined conditions. Interestingly, AiNPCs had robust expression of regional marker genes for forebrain but not for midbrain or hindbrain. Therefore, the AiNPCs were more readily differentiated into glutamatergic and GABAergic neurons, but not dopaminergic neurons. However, overexpression of Lhx8 and Foxa2/Lmx1a in AiNPCs promoted cholinergic and dopaminergic neuronal differentiation, respectively, suggesting that fate-committed AiNPCs can be shifted to other lineages through forced expression of specific TFs.

To date, various cell sources has been used to generate iNPCs, including fibroblasts, astrocytes, sertoli cells, and urine cells. The successful conversion of different types of somatic cells into iNPCs suggests a common iNPCs reprogramming path. Our current study suggests the same NPC transcriptional core network, used for mouse fibroblast reprogramming, can superimpose a NPC fate onto astrocytes [19]. Given the neural origin of astrocytes, it is possible that fewer TFs or even a single TF may be able to coerce astrocytes into the same NPC fate [49]. Cultured astrocytes are quite heterogeneous and may manifest a spectrum of phenotypes for NPC conversion. In specific culture conditions, developing or adult damaged brains might contain immature or reactive astrocytes, respectively, that exhibit neurosphere-forming ability [30, 50]. In our studies, the astrocytes are likely not in reactive status because we did not observe any EGFP^+^ neurospheres generated from the control astrocyte cultures even though growth factors were present. Furthermore, the protocols to generate reactive astrocytes usually need physical damage or chemical stimulation, which is not used in our culture conditions [51, 52]. We have previously activated the culture to generate a reactive phenotype, suggesting a lower activation status of our astrocyte culture [53]. It is possible that retroviral transduction diminished the efficiencies for NPC conversion. In addition, astrocyte cultures in our studies are derived from cortices of P7 mice, which are likely quite different from adult astrocytes. Caution needs to be taken to translate the findings to the adult brain and to the in vivo experiments.

Our study also confirm that Foxg1, Sox2, and Brn2 may be one of the master regulator sets for stabilizing the status of NPC, given that NPCs stand for a progenitor population with diverse fate restrictions in a region-specific manner [54, 55]. We cannot exclude the possibility that neuronal differentiation potential of AiNPCs could be altered by extended passages, where cell fate may be modulated by endogenous machinery. Nonetheless, throughout all AiNPCs passages, the AiNPCs manifested all cardinal properties of definitive NPCs: proliferating, self-renewal, tripotency and most importantly, a specific regional identity.

iNPCs theoretically could generate all known neuronal subtypes in the brain. Surprisingly, we found that AiNPCs are committed to a clear regional fate with the 3 factors-imposed transcriptional core network. The telencephalic fate commitment may be largely due to the inclusion of Foxg1, a critical denominator of ventral telencephalic fate during development. It starts to express when the anterior part of the neural plate is specified to telencephalon (E8.5), after the emergence of primitive NPCs at E7 [56–58]. NPCs interpret patterning cues of neuronal specification within a very short window (E8.5 to E10.5) at early stages of neural development [29] and Foxg1, whose expression is independent of SHH [59], acts autonomously to endow cells with intrinsic competency to obtain ventral telencephalic identity [57].

Our neuronal subtype specification studies suggested the spontaneous differentiation of AiNPCs is biased to favor glutamatergic/GABAergic neuronal differentiations. This trend follows a similar neural development program in vivo, that NPCs in the dorsal and ventral telencephalon majorly develop into glutamatergic and GABAergic neurons during brain development [60]. Similar patterns were also observed when differentiate telencephalic NPCs in vitro, confirming the differentiation preference of telencephalic NPCs bias to glutamatergic and GABAergic neurons [61]. Besides, a small proportion of cholinergic neurons could be differentiated from AiNPCs, whereas no dopaminergic neuron generation takes place, which further confirmed the telencephalic phenotype of AiNPCs. Our results suggested that the generation of other types of neurons from AiNPCs involves more regional-/subtype-specific TFs. Lhx8, a member of the LIM homeobox gene family, is selectively expressed in the medial ganglionic eminence [62]. Lhx8-null mice presented significantly less basal forebrain cholinergic neurons [63]. In our studies, significant increase in the generation of ChAT^+^ cholinergic neurons was found in Lhx8-overexpressed AiNPCs. These results confirm that Lhx8 positively regulates cholinergic differentiation. However, the efficiency of cholinergic neuron generation is still relatively low even with Lhx8 overexpression. For further enhancement of cholinergic neurons differentiation, approaches that modify the microenvironment of AiNPCs might be recruited, such as small molecule treatment, astrocyte co-culture and 3D scaffold culture, which are under investigation.

Foxa2 & Lmx1a are reported to determine the cell fate of dopaminergic neurons in midbrain cooperatively [64, 65]. We previously reported that a defined TFs set including Foxa2 could reprogram mouse fibroblasts into dopaminergic precursors efficiently [23]. Here, we demonstrated that Foxa2/Lmx1a could overwrite the telencephalic fate commitment of AiNPCs and direct the differentiation of AiNPCs into a mesodiencephalic dopaminergic fate. Interestingly, the influence of Foxa2 on the induction of DAs differentiation is significantly stronger than that of Lmx1a in our studies, suggesting Foxa2 plays its role more upstream and may regulate multiple downstream factors besides Lmx1a. Similar findings were reported that during the mouse midbrain development in vivo, Foxa family members (Foxa1 & Foxa2) function upstream of Lmx1a/b, together with other dopaminergic neuron determinant genes such as Nkx2.2 and TH to promote mesodiencephalic DA differentiation [65]. Lmx1a can only specify mesodiencephalic dopaminergic fate within Foxa2^+^ mesencephalic progenitors [66]. Thus, our and other independent groups’ observations demonstrate the key gene network for the cell fate determination of DAs.

The reprogramming of somatic cells into proliferative iNPCs is a promising technique to prepare sufficient cells for cell replacement purpose. We previously reported the survival and maturation of reprogrammed dopaminergic precursors in MPTP-treated mouse brain. AiNPCs and their-derived cell-type specific precursors may broaden the precursor cell types suitable to be transplanted in neurodegenerative disease mouse models. These cells are not only able to achieve therapeutic effects through cell replacement, but also to modulate brain microenvironment for tissue repair and regeneration [67, 68]. Thus, the effects of AiNPCs transplantation and the underlying in vivo mechanisms remain open propositions, which is currently under investigation.

## Conclusions

**Fig. 7:**
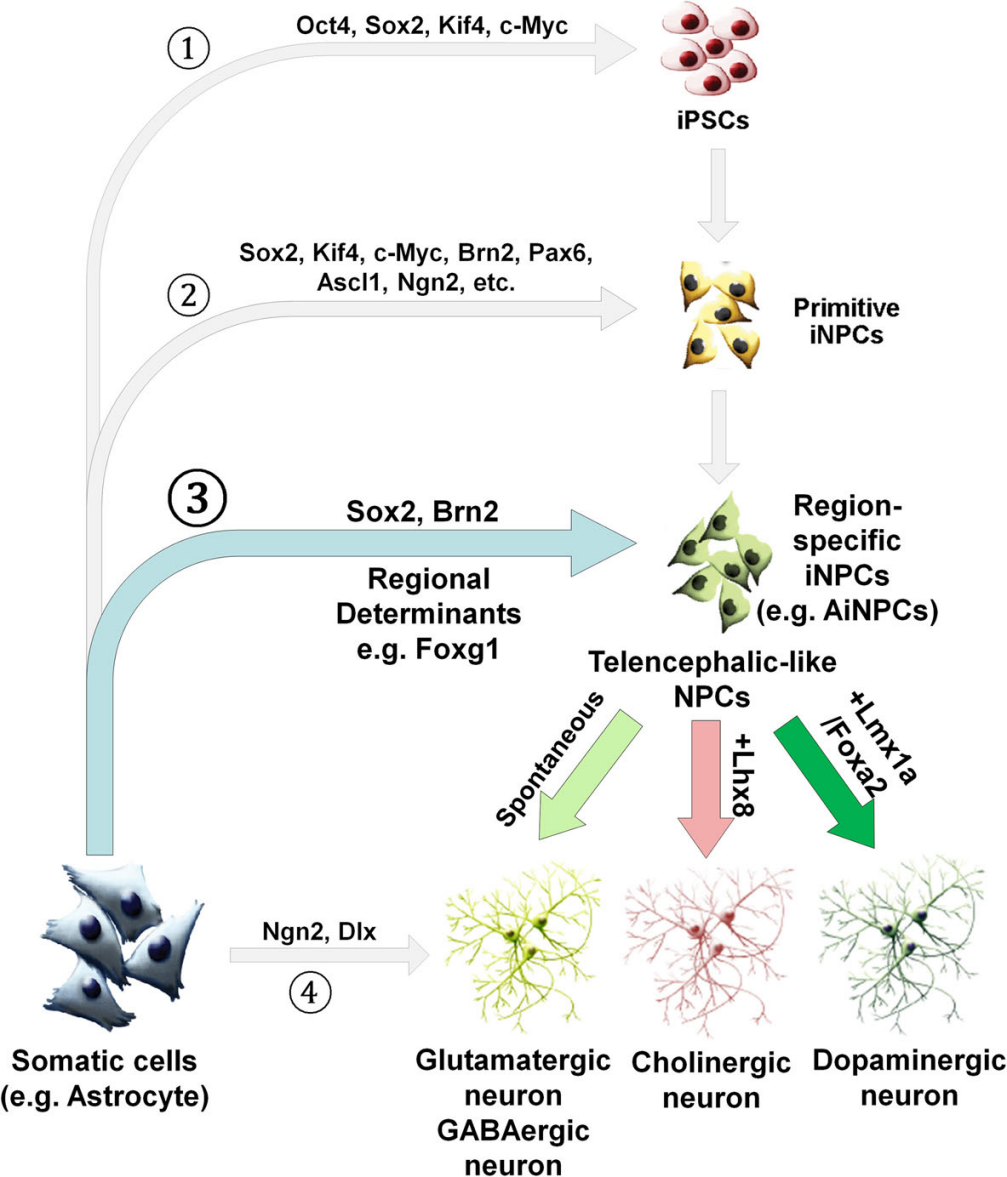
Proposed model for reprogramming for iNPCs and differentiation for specific lineages of neurons. Combination of Yamanaka Factors (Oct4, Sox2, Klf4, c-Myc) and ESC media leads to iPSC dedifferentiation from somatic cells (Path 1). Direct conversion of somatic cells into iNPCs was achieved by transducing fibroblasts or astrocytes with NPC fate determinants (Path 2). Induced neurons are termed after terminally differentiated cells were directly converted into neurons (Path 4). We propose that somatic cells can be converted to regional-committed iNPCs with restricted neuronal subtype differentiation capacities (e.g. glutamatergic and GABAergic neurons for telencephalon). The transdifferentiation is achieved using NPC fate master regulators (e.g., Sox2 and Brn2) in addition to potent regional determinants (e.g. Foxg1 for telencephalon) (Path 3). The differentiation potential of AiNPC differentiation towards forebrain Gluamatergic and GABAergic neurons can be altered by the forced expression of TFs that promotes dedicated neuronal specifications (e.g., Lhx8 for cholinergic neurons and Foxa2/Lmx1a for midbrain dopaminergic neurons)

In summary, astrocyte is a promising candidate for iNPC direct conversion. Targeting astrocytes with specific viral-based transgene delivery to induce NPC fate is particularly attractive since it is able to target those pre-existing reactive astrocytes within the proximity of lesion areas and may have great therapeutic potentials in neurodegenerative diseases. Furthermore, Foxg1, Sox2, and Brn2 may be one of the master regulator sets for successfully superimposing NPC fate not only on fibroblasts but on astrocytes as well (Fig. 7). The AiNPCs demonstrate cardinal features of NPCs with ventral telencephalic identity and differentiation capacity. The limitations in differentiation potentials of AiNPCs could be overcome by factors promoting dedicated neuronal specifications. Because reactive astrogliosis is ubiquitous in neurodegenerative disorders, our results suggest astrocyte-iNPC reprogramming is a potentially promising strategy to boost brain regeneration. The identification of specific factors for neuronal subtype generation could offer valuable information for cell-based therapy for devastating neurodegenerative diseases.

## Additional file

Additional file 1: **Figure S1.** The validation of primary astrocyte culture. A-C: Primary astrocyte cultures were positive for GFAP, but negative for Iba1 and Tuj1. Scale bars represent 20 μm. **Figure S2.** The validation of transcription factors transduction. A, B: The transduction was validated by examining the expression of endogenous (A) and exogenous (B) Sox2, Brn2, and Foxg1. Error bars denote s.d. from triplicate measurements. **Figure S3.** The methylation status of SSEA-1 promoter. The SSEA-1 promoter regulatory region DNA methylation patterns of AiNPCs, control astrocytes, control NPCs and astrocyte-derived iPSCs were analyzed using pyrosequencing method. *Human lymphocyte genomic DNA was used as a negative control for pyrosequencing. **Sss1 methyltranferase treated human lymphocyte genomic DNA was used for positive control. **Figure S4.** The effects of Lhx8 forced expression in the differentiation of neuronal subtypes from AiNPCs. A. The generation of VGLUT^+^ glutamatergic neurons, GABA^+^/Darpp32^+^ GABAergic neurons and TH^+^ dopaminergic neuron after transduction of Lhx8 in AiNPCs were determined by immunocytochemistry. B. The percentage of different subtypes of neurons was quantified by counting VGLUT^+^, GABA^+^, Darpp32^+^ and TH^+^ cells and comparing against the total number of cells. Scale bars represent 50 μm (A). Error bars denote s.d. from triplicate measurements (B). **Table S1.** Antibody List. **Table S2.** Primers for Marker Genes. **Table S3.** Pyrosequencing Primer Sequences. (DOCX 4248 kb)

## Abbreviations

AD: Alzheimer’s disease
AiNPCs: Astrocyte-derived induced neural progenitor cells
CNS: Central nervous system
iNPCs: Induced neuronal progenitor cells
iPSCs: Induced pluripotent stem cells
NPCs: Neural progenitor cells
PD: Parkinson’s disease
TFs: Transcription factors
