# Supplementary material for: Direct conversion of mouse astrocytes into neural progenitor cells and specific lineages of neurons
Source: Transl Neurodegener. 2018 Nov 5;7:29. doi: 10.1186/s40035-018-0132-x (PMC6217767; doi:10.1186/s40035-018-0132-x)
Supplement: Supplementary file 1 — Figure S1. The validation of primary astrocyte culture. A-C: Primary astrocyte cultures were positive for GFAP, but negative for Iba1 and Tuj1. Scale bars represent 20 μm. Figure S2. The validation of transcription factors transduction. A, B: The transduction was validated by examining the expression of endogenous (A) and exogenous (B) Sox2, Brn2, and Foxg1. Error bars denote s.d. from triplicate measurements. Figure S3. The methylation status of SSEA-1 promoter. The SSEA-1 promoter regulatory region DNA methylation patterns of AiNPCs, control astrocytes, control NPCs and astrocyte-derived iPSCs were analyzed using pyrosequencing method. *Human lymphocyte genomic DNA was used as a negative control for pyrosequencing. **Sss1 methyltranferase treated human lymphocyte genomic DNA was used for positive control. Figure S4. The effects of Lhx8 forced expression in the differentiation of neuronal subtypes from AiNPCs. A. The generation of VGLUT+ glutamatergic neurons, GABA+/Darpp32+ GABAergic neurons and TH+ dopaminergic neuron after transduction of Lhx8 in AiNPCs were determined by immunocytochemistry. B. The percentage of different subtypes of neurons was quantified by counting VGLUT+, GABA+, Darpp32+ and TH+ cells and comparing against the total number of cells. Scale bars represent 50 μm (A). Error bars denote s.d. from triplicate measurements (B). Table S1. Antibody List. Table S2. Primers for Marker Genes. Table S3. Pyrosequencing Primer Sequences. (DOCX 4248 kb) [file 40035_2018_132_MOESM1_ESM.docx]

**S1**


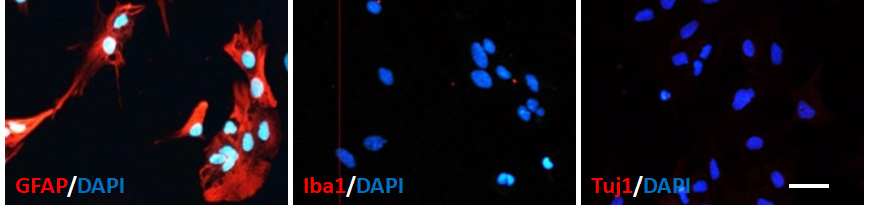


**Figure S1.** **The validation of primary astrocyte culture. A-C:** Primary astrocyte cultures were positive for GFAP, but negative for Iba1 and Tuj1. Scale bars represent 20 µm.

**S2**


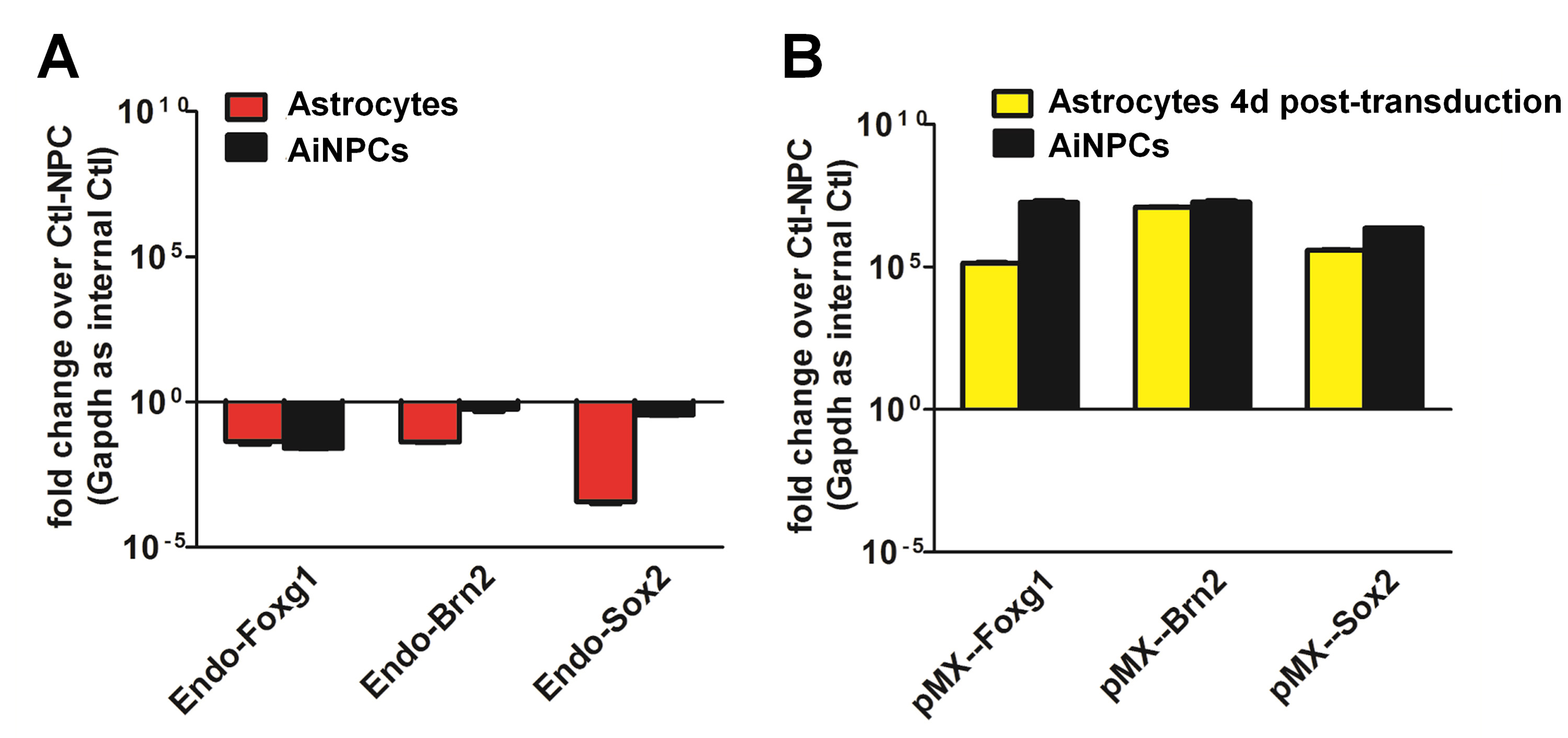


**Figure S2.** **The validation of transcription factors transduction. A, B:** The transduction was validated by examining the expression of endogenous (A) and exogenous (B) Sox2, Brn2 and Foxg1. Error bars denote s.d. from triplicate measurements.

**S3**


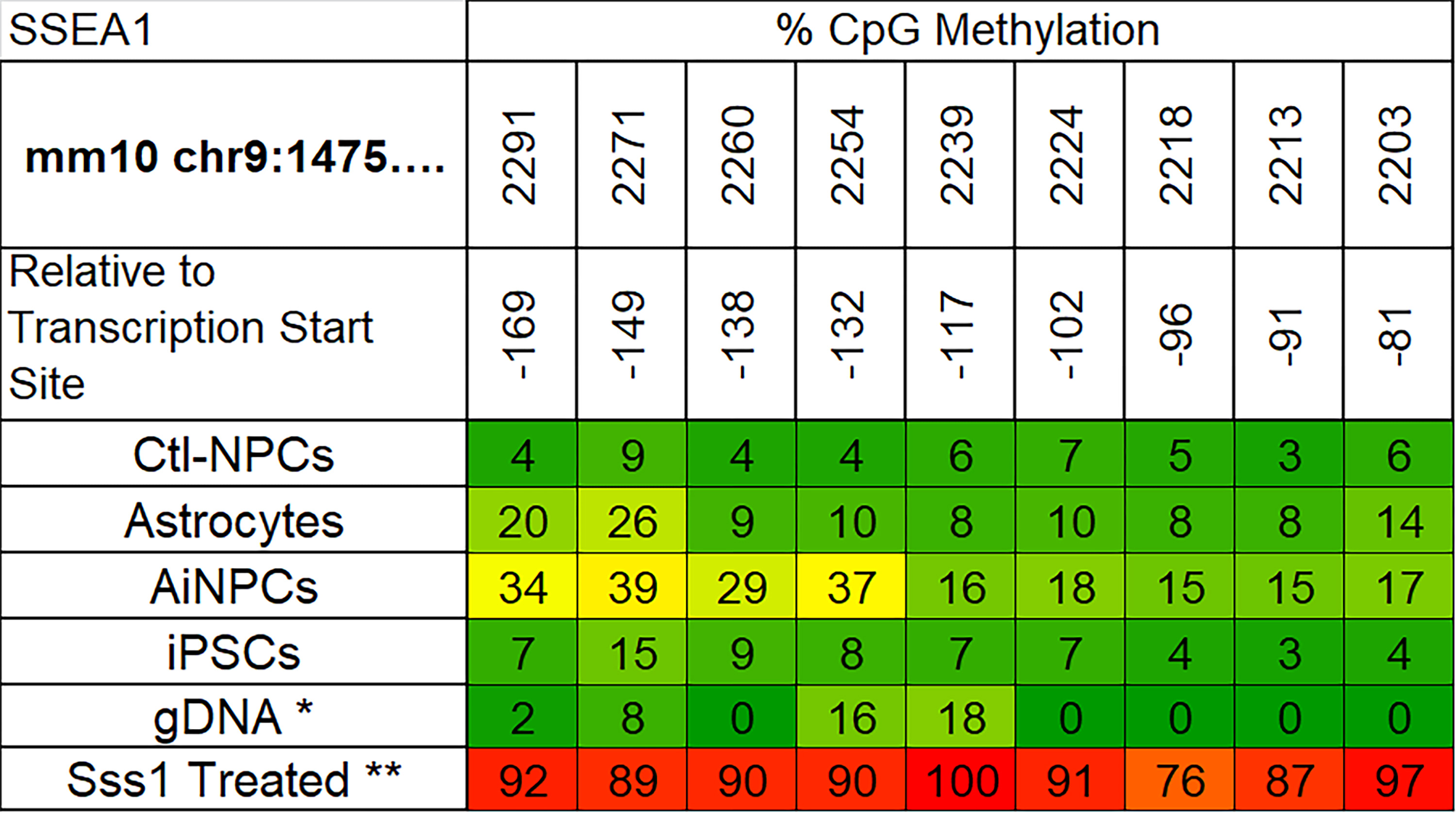


**Figure S3.** **The methylation status of SSEA-1 promoter.** The SSEA-1 promoter regulatory region DNA methylation patterns of AiNPCs, control astrocytes, control NPCs and astrocyte-derived iPSCs were analyzed using pyrosequencing method. *Human lymphocyte genomic DNA was used as a negative control for pyrosequencing. **Sss1 methyltranferase treated human lymphocyte genomic DNA was used for positive control.

**S4**


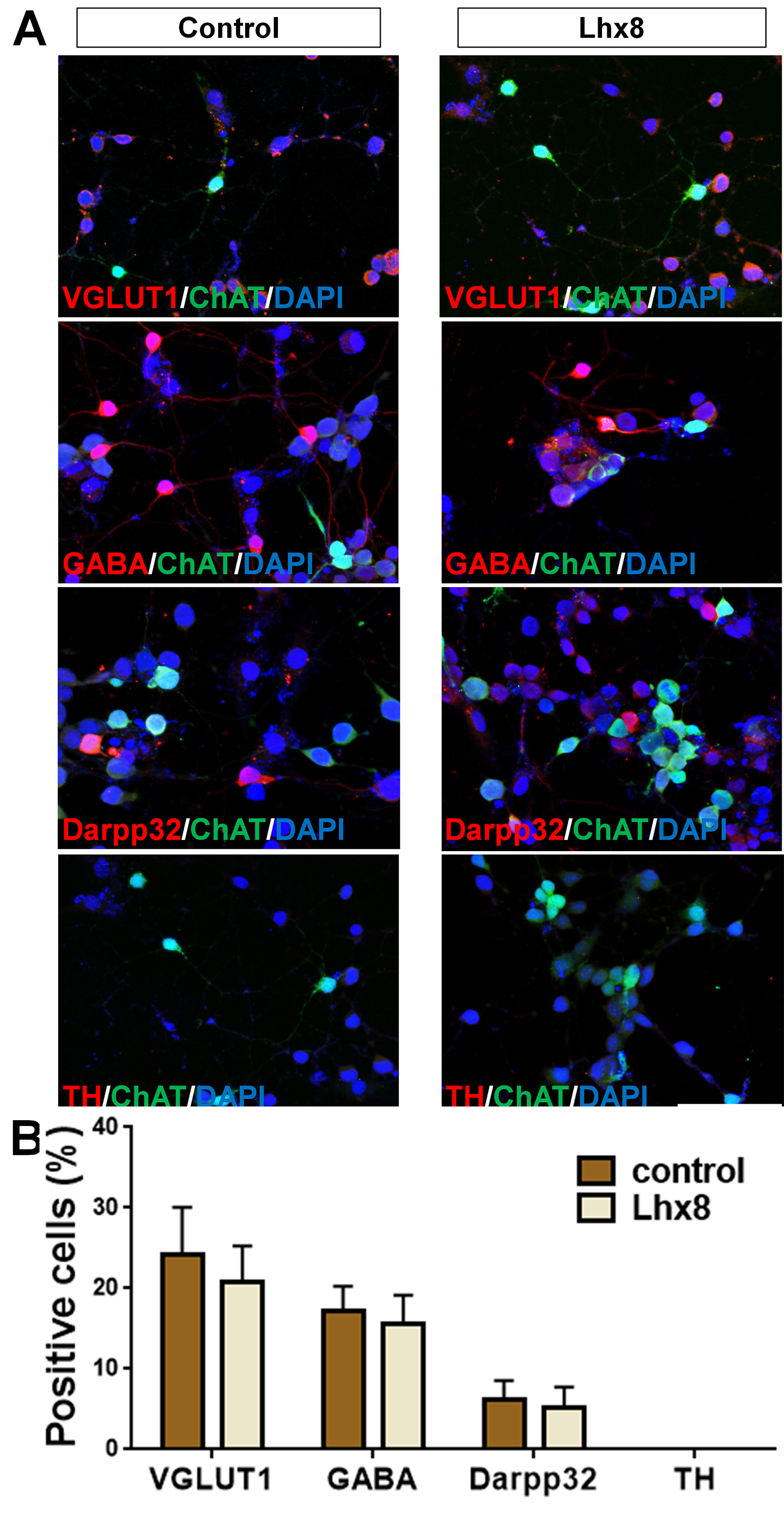


**Figure S4.** **The effects of Lhx8 forced expression in the differentiation of neuronal subtypes from AiNPCs. A.** The generation of VGLUT^+^ glutamatergic neurons, GABA^+^/Darpp32^+^ GABAergic neurons and TH^+^ dopaminergic neuron after transduction of Lhx8 in AiNPCs were determined by immunocytochemistry. **B**. The percentage of different subtypes of neurons was quantified by counting VGLUT^+^, GABA^+^, Darpp32^+^ and TH^+^ cells and comparing against the total number of cells. Scale bars represent 50 µm (A). Error bars denote s.d. from triplicate measurements (B).

**Table S1. Antibody List**

| **Antibody** | **Isotype** | **Dilution** | **Source** |
| --- | --- | --- | --- |
| ChAT | Goat IgG | 1:300 | Millipore |
| GABA | Mouse IgG | 1:1,000 | Sigma |
| GFAP | Rabbit IgG | 1:1,000 | DAKO |
| MAP2 | Rabbit IgG | 1:1,000 | Sigma |
| Nestin | Mouse IgG1 | 1:1000 | DSHB |
| O4 | Mouse IgG | 1:100 | R&D Systems |
| Sox2 | Rabbit IgG | 1:1500 | abcam |
| Synaptophysin | Mouse IgG1 | 1:1,000 | abcam |
| Tau | Rabbit IgG | 1:200 | abcam |
| TH | Rabbit IgG | 1:2,000 | Cal Biochem |
| Tuj | Mouse IgG | 1:1500 | Sigma |
| Tuj | Rabbit IgG | 1:1500 | Sigma |

**Table S2. Primers for Marker Genes**

| **Gene** | **Forward Primer** | **Reverse Primer** |
| --- | --- | --- |
| Aldh1l1 | GAGTGTGACGTGCTTCCAGA | TGGTCCCAGTTGATCATGGC |
| Aqp4 | CAGCATCGCTAAGTCCGTCT | CGTTTGGAATCACAGCTGGC |
| Blbp | GGACACAATGCACATTCAAGAAC | CCGAACCACAGACTTACAGTTT |
| CD44 | TCTGCCATCTAGCACTAAGAGC | GTCTGGGTATTGAAAGGTGTAGC |
| Darpp32 | AAGAGGATGAGTTAGGGGAGC | GCCACAAGTAGGCTTCTGC |
| Emx2 | TAAAAGTATGGTTTCAGAACCGGAGAAC | TGTTGAGAATCTGAGCCTTCTTCCTC |
| Fgfr3 | TGGATCAGTGAGAATGTGGAGG | CCTATGAAATTGGTGGCTCGAC |
| Gad65 | TGCTTCAGTACGTGGTGAAAAG | TCCTCCAGATTTTGCGGTTGG |
| Gbx2 | CCCTCAAACTCAGCGAGGTGC | GCGAACCTGCTAACGTGAACAGG |
| GFAP | CTGGAACAGCAAAACAAGGCGCTGG | TCCAGCCTCAGGTTGGTTTCATC |
| Glast | ACCAAAAGCAACGGAGAAGAG | GGCATTCCGAAACAGGTAACTC |
| Gpm6a | GCTTGGACAAGTGGACTGC | TAGGGAATACCTCCCAGGCA |
| Hoxb2 | ATTCGCCTTTTCTACCGGACC | GGGCTATCGAGAGAACCCTG |
| Igfbp3 | CCAGGAAACATCAGTGAGTCC | GGATGGAACTTGGAATCGGTCA |
| Mash1 | GCAACCGGGTCAAGTTGGT | GTCGTTGGAGTAGTTGGGGG |
| Msi1 | GATGGCTCCCCCTCCAGGTT | CATTGGTGAAGGCTGTGGCA |
| Nanog | TCTTCCTGGTCCCCACAGTTT | GCAAGAATAGTTCTCGGGATGAA |
| Ncan | GCACCGTGTATGGCTGTAGT | ATTCTCGCAAGGGCTGCATA |
| Nestin | GTCTCAGGACAGTGCTGAGCCTTC | TCCCCTGAGGACCAGGAGTCTC |
| Oct4 | GGCTTCAGACTTCGCCTTC | AACCTGAGGTCCACAGTATGC |
| Otx2 | TATCTAAAGCAACCGCCTTACG | AAGTCCATACCCGAAGTGGTC |
| S100b | GAGCTGGAGAAGGCCATGGT | AAGGCCATGAACTCCTGGAAGTCA |
| Sox1 | GAGATGATCAGCATGTACCTGCC | GTAGTGCTGTGGCAGCGAGT |
| Sox2 | CCTCCGGGACATGATCAGCATGTA | GCAGTGTGCCGTTAATGGCCGTG |
| Sox3 | CAGCTCGAGAGAACGCATCA | ACGGGGTTCTTGAGTTCAGT |
| SSEA-1 | ACGGATAAGGCGCTGGTACTA | GGAAGCCATAGGGCACGAA |
| TH | GTGCCAGAGAGGACAAGGTTC | CGATACGCCTGGTCAGAGA |
| Tox3 | CTGGGGTACTACGGCTACAG | CGTGTGGAATGTCTGCTCACT |
| Zbtb16 | CTGGGACTTTGTGCGATGTG | CGGTGGAAGAGGATCTCAAACA |
| Zfp42 | CCCTCGACAGACTGACCCTAA | TCGGGGCTAATCTCACTTTCAT |

**Table S3. Pyrosequencing Primer Sequences**

| **Gene** | **Forward Primer** | **Reverse Primer** | **Sequencing Primer** | **Annl °C** |
| --- | --- | --- | --- | --- |
| Nestin | AGTGTTTATGTTAGATTTTAGGGATAT | AACAAAAACTAAACAAAACCAATAACA | GTTAGATTTTAGGGATATTTG | 60 |
| Oct4 | GAGGATGGTTGAGTGGGTTGTAAGGAT | CTTCAAAATCCTCTCACCCCTACCTTA | GTTGAGTGGGTTGTAAGGATAG | 60 |
